# Principles of Regulation of Self-Renewing Cell Lineages

**DOI:** 10.1371/journal.pone.0072847

**Published:** 2013-09-03

**Authors:** Natalia L. Komarova

**Affiliations:** Department of Mathematics, University of California Irvine, Irvine, California, United States of America; UC Santa Barbara, United States of America

## Abstract

Identifying the exact regulatory circuits that can stably maintain tissue homeostasis is critical for our basic understanding of multicellular organisms, and equally critical for identifying how tumors circumvent this regulation, thus providing targets for treatment. Despite great strides in the understanding of the molecular components of stem-cell regulation, the overall mechanisms orchestrating tissue homeostasis are still far from being understood. Typically, tissue contains the stem cells, transit amplifying cells, and terminally differentiated cells. Each of these cell types can potentially secrete regulatory factors and/or respond to factors secreted by other types. The feedback can be positive or negative in nature. This gives rise to a bewildering array of possible mechanisms that drive tissue regulation. In this paper, we propose a novel method of studying stem cell lineage regulation, and identify possible numbers, types, and directions of control loops that are compatible with stability, keep the variance low, and possess a certain degree of robustness. For example, there are exactly two minimal (two-loop) control networks that can regulate two-compartment (stem and differentiated cell) tissues, and 20 such networks in three-compartment tissues. If division and differentiation decisions are coupled, then there must be a negative control loop regulating divisions of stem cells (e.g. by means of contact inhibition). While this mechanism is associated with the highest robustness, there could be systems that maintain stability by means of positive divisions control, coupled with specific types of differentiation control. Some of the control mechanisms that we find have been proposed before, but most of them are new, and we describe evidence for their existence in data that have been previously published. By specifying the types of feedback interactions that can maintain homeostasis, our mathematical analysis can be used as a guide to experimentally zero in on the exact molecular mechanisms in specific tissues.

## Introduction

Tissue homeostasis is key to the functioning of multi-cellular organisms, and an understanding of the mechanisms involved in tissue regulation is not only crucial from a basic biological perspective, but also from a human health perspective. The emergence of cancer requires escape of cells from homeostatic control, resulting in the selfish and unrestrained growth of cells. Feedback loops are thought to play a central role for achieving homeostatic control. This notion is supported by a variety of experimental findings. Negative feedback regulation affecting the processes of cell division and differentiation has been documented in the mouse olfactory epithelium, involving the regulatory proteins GDF11 and activin [1], [2]. Similarly, evidence for feedback regulation has been found in other tissues such as skeletal muscle, bone, keratinocytes, and the hematopoietic system, identifying specific regulatory proteins that mediate the feedback in each case [3]–[7]. Further evidence comes from the study of human cancers where feedback regulatory mechanisms are disrupted. The transforming growth factor beta (TGF-beta) is an important regulator in many tissues. A range of cancers circumvent TGF-beta growth inhibition by inactivating the genes for the TGF-beta receptors or through downstream alterations that disable the tumor-suppressive arm of the pathway [8]–[10]. Colorectal cancer involves the loss of the APC gene and the consequent activation of the Wnt cascade, followed by the activation of the K-Ras oncogene [11], changes that again disable feedback regulatory processes. Another example is bone morphogenic protein 4 pathway (BMP4), which can regulate the patterns of division and differentiation in human glia cells and which is silenced in glioblastomas [12].

These data make it evident that feedback regulatory processes play a major role in tissue homeostasis and that they need to be overcome in proliferative diseases such as cancer. Despite this wealth of data, there is less understanding of the exact mechanisms that underlie feedback regulation. It is often unclear which cells in the lineage secrete regulatory factors and which cells respond. Typically, tissue contains the stem cells, transit amplifying cells and terminally differentiated cells. Each of these cell types can potentially secrete regulatory factors and/or respond to them. The feedback can be positive or negative in nature, i.e. having a weaker or a stronger signal can increase or decrease the overall probability of a cellular fate decision in a given compartment. This gives rise to a bewildering array of possible mechanisms that drive tissue regulation. Identifying the exact regulatory circuits that can stably and robustly maintain tissue homeostasis is critical for our basic understanding of multicellular organisms and is equally critical for identifying how tumors circumvent this regulation, thus providing targets for treatment. While molecular approaches are undoubtedly making great strides in this respect, the vast array of possible mechanisms renders this task very difficult. Here we use mathematical models to narrow down the possibilities in this search. Not all feedback interactions that are potentially possible in cell lineages are capable of maintaining tissue homeostasis. In fact, many fail to do so. Moreover, different feedback circuits are characterized by different degrees of robustness and stability. The mathematical analysis can specify the types of feedback interactions that can indeed maintain homeostasis, and this can be used as a guide to experimentally zero in on the exact molecular mechanisms in specific tissues. For example, the mathematical analysis can identify the minimal number of control loops required for homeostasis, which cells in the lineage need to produce regulatory factors and which have to respond, whether specific types of loops need to involve positive or negative feedback, and how the types of required regulatory mechanisms depend on the number of cell compartments in the lineage.

Stem cell regulation is often described in the context of the so-called stem cell niche, an anatomic location that regulates how stem cells participate in tissue generation, maintenance and repair [13]. The niche includes both cellular and non-cellular components that interact in order to control the adult stem cell [14]. Within a niche, the stem cell fate - that is, its division and differentiation decisions - are under the regulation of many different factors, including structural and physical forces, paracrine and endocrine signaling from neighboring and distant cells, metabolic factors and neural signaling, see also [15]. The number of stem cells is maintained under signaling from the stem cell population to itself, from surrounding and distant daughter cells, and from various components of the stem cell niche, including the endothelium, pericites, and surrounding extracellular matrix. Many different regulatory mechanisms have been discussed, including growth factors, cell-cell contacts, and cell-matrix adhesions [16], regulation by microRNAs [17], [18], signaling from mesenchymal cells, as well as differentiated cells [19]. In [14], both physical contact with the niche, and diffusible factors that regulate stem cell behavior, have been cataloged for neural, epidermal, haematopoietic, and intestinal stem cells. Many more mechanisms exist that are responsible for controlling cell decisions of both stem cells and other cell types, see e.g. [20]–[36], and a more detailed review later in this paper. Is each regulation mechanism unique, or can we find patterns and common motives of regulation across different tissues?

Several insightful theoretical studies have been published on control dynamics of biological networks [37]–[40] and stem cell regulation [41]–[45], e.g. in relation to carcinogenesis [46]–[51], and in haematopoietic system [52]–[54]. Important steps in our understanding of the underlying general principles and logic of cell lineage control have been made recently [1], [55]–[57]. In these papers, a novel way of reasoning about stem/daughter cell regulation has been proposed, which looks to re-evaluate the biological data on signaling pathways and understand the design principles of renewing tissues from an engineering prospective. While many specific factors and gene products are being identified to play a role in cell fate decisions, it is important to look at the self-renewing system as a whole, and study the topology of the regulatory networks orchestrating tissue renewal. Specifically, it is important whether the regulatory factors are produced by the same cell type they act upon, or by a different compartment, whether it is the more differentiated cells downstream, or the less differentiated cells upstream. What are the possible geometries of inter- and intra-compartment control loops that can ensure stable homeostasis? How many loops are needed for successful maintenance of a steady population? Should they be positive or negative? What are the minimal controls still compatible with stability?

In this paper we pursue these questions and uncover the number and types of different regulation mechanisms that are compatible with (1) steady state maintenance, (2) fluctuation control, and (3) system robustness. Our study differs from the previous literature because we do not make any a priory assumptions on the type and direction of signaling loops. Instead, we investigate the populations dynamics of cell lineages in the most general setting and single out the numbers and the types of control loops that are capable of robustly maintaining stability. All the building blocks of the control mechanisms that we uncover are consistent with observations in various systems. Some of the control mechanisms have been investigated/hypothesized previously. Many are proposed for the first time.

## Results

### The Framework

Our model keeps track of the total populations of cells in different compartments: stem cells, intermediate cells such as transit amplifying cells, and terminally differentiated cells. There can be 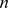 compartments in the lineage. All cell types, except for the terminally differentiated cells, are capable of dividing at a rate 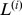 (per population), where index 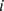 marks the cell type. Terminally differentiated cells die at a rate 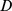. Divisions are symmetric, and two possibilities exist. With probability 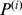 two daughter cells are produced, which belong to the next differentiation stage; this represents the process of differentiation. With probability 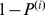, two cells are produced which are identical to the dividing cell, which represents the process of cell proliferation. This general model is conceptually close to the models proposed in [1].

**Figure 1:**
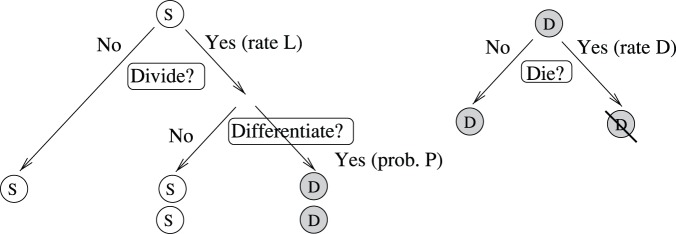
The schematic of cellular decisions and regulation by cell populations. The circles marked with “S” and “D” denote stem and differentiated cells respectively. On the left, a stem cell decision tree is shown, which includes division/senescence decisions as well as proliferation/differentiation decisions. On top right, a differentiated cell decision tree is shown. All the decisions can be controlled by factors produced by the stem cell population and/or differentiated cell population. The control can be negative or positive in each case.

Which cell divides when and what division type it undergoes must be subject to feedback loops, to assure that the populations sizes do not deviate dangerously from the physiologically desired levels, as a result of random fluctuations [58]. The framework adopted here is depicted schematically in figure 1. It shows the example of a simplified system where only two cell types are present: stem cells and differentiated cells. Each stem cell faces two types of decisions: division/senescence decisions, where it divides with rate 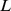, and (upon division) proliferation/differentiation decisions, where it differentiated with probability 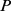. Daughter cells die with rate 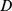. All the decisions can be controlled by factors produced by the stem cell population and/or differentiated cell population, which is shown schematically by thick arrows. Mathematically this is reflected in the fact that quantities 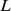, 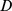, and 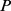 are not constant parameters, but can be functions of the population sizes of different compartments.

In the literature, several different functional forms have been implemented [1], [46]–[51], [58], [59], and the results of these assumptions investigated. In the present paper we do not attempt to assume a specific functional form of these control mechanisms. The functional forms are not known at the present stage. Instead, we take a different approach. It turns out that a lot of insights can be obtained by simply examining (i) the topology of control (that is, which populations control which of the processes), and (ii) the sign of the control (that is, whether it is a positive or negative loop).

The first example comes from a two-compartment system consisting of stem cells (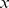) and differentiated cells (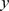). Empirical deterministic equations describing the population maintenance are given by
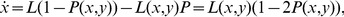
(1)

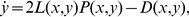
(2)where superscripts 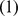 have been dropped; please see Materials and Methods for a fully stochastic description. The steady state is characterized by populations of size 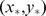 with



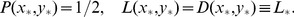
(3)
Equation (3) states that at equilibrium, the probability to proliferate equals to the probability to differentiate, and the rate of divisions equals the rate of death.

Let us denote by 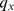 and 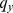 the partial derivatives of the net growth rate, 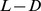, with respect to 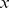 and 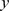 (divided by 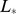). Further we denote by 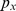 and 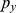 the partial derivatives of the differentiation probability, 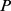, with respect to 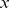 and 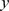. To clarify the biological meaning of these parameters, consider the quantity 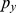. If it is nonzero, it means that the probability of stem cell differentiation is controlled by the differentiated cell population. Moreover, if 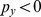, this means that the control is negative (the more differentiated cells in the system, the less likely the stem cells are to differentiate); 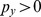 means the existence of a positive control loops. The other three quantities can be interpreted in a similar manner.

It turns out that much of the system’s behavior is independent of the particular functional forms of 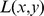, 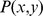 and 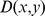, and only the four numbers matter: 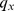, 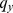, 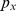, and 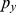. In particular, stability conditions of the system are given by two inequalities:
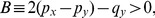
(4)

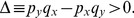
(5)


Further, the amounts of fluctuations in the two populations are given by
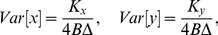
where constants 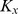 and 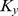 can be found in the Materials and Methods. Finally, the magnitude of the quantity 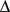 in equation (4) defines the system robustness with respect to parameter changes (the larger 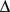, the higher system robustness).

It is clear that the key features of the stem cell system are contained in the numbers 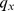, 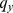, 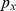, and 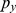, which we can simply call “controls”. In particular, the topology of the network is defined by which of these numbers are nonzero, and the signs of controls correspond to the signs of these four factors. Next, we examine biological literature to find what is known about these control factors.

### Different Types of Control

#### Cell numbers negatively control differentiation


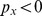
, 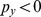. Crowding and factors like contact inhibition play an important role in determining the fate of stem cells. Cell shape (rounded or flat), as well as mechanical stress received from surrounding cells, control proliferation and differentiation decisions [30]. In particular, it is well known that more rounded cells tend to differentiate, while flattened cells retain stem-cell properties in vitro. Mechanic strain has been shown to inhibit differentiation [23], [28]. This suggests that crowding inhibits differentiation, that is, the number of cells negatively affects the differentiation probability.

#### Cell numbers positively control differentiation


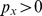
, 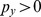. Extracellular signals and the micro-environment constitute a niche, in which stem cells compete for limiting concentrations of growth factors, thereby maintaining a balance between self-renewal and differentiation. Wnt protein has been shown to promote stem cell self-renewal [60]. If such self-renewal-promoting factor is secreted by the cells of the stem cell niche, then the stem cells in the proximity of the source of the signal will tend to self-renew, while stem cells further away will tend to differentiate. This corresponds to a positive control of differentiation by the stem cells (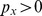): the more stem cells there are, the more likely it is that a given stem cell will find itself relatively far from the niche, and thus its probability to differentiate will increase.

Furthermore, in some systems, mechanical strain has been shown to increase cell differentiation [21], [29], [30], leading to the same trend: 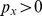, 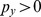.

Finally, it has been suggested that stem cells have to be spatially localized to their niches, which keeps them protected from the differentiating influences of the surrounding microenvironment [25]. Therefore, as the number of stem cells increases, the probability to be exposed to the differentiation signals from the outside increases, resulting in a positive control loop.

#### Control of differentiation from downstream


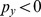
. In the context of the adult neurogenesis, it has been proposed that once generated, neural stem cell descendants can trigger some type of feedback mechanism to stop stem cell differentiation [20]. Notch signaling has been considered a candidate to regulate such a feedback mechanism during adult neurogenesis [22]. Another similar mechanism is provided by Prox1 expression [31], which links adult neural stem cell self-maintenance with the generation of the proper number of descendants. In both cases there seems to be a negative regulation of differentiation from downstream, 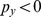.

In the colon, signals acting on intestinal stem cells (ISC) are derived from mesenchymal cells, as well as differentiated intestinal epithelial cells [19]. Members of the Wnt/Wingless family appear to reside at the center of the signaling network that promotes ISC renewal. As in neurogenesis, Notch signaling also plays an important role in intestinal stem cell regulation, controlling the balance between self renewing stem cells and their differentiating progeny [19].

In [32] it is reported that haematopoietic stem cells are regulated by their mature progeny. A feedback loop is described in which platelet numbers, through regulation of available thrombopoietin levels, regulates the entry of haematopoietic stem cells into cycle.

In [1] it is reported that in the olfactory epithelium, differentiated cells (olfactory receptor neurons) produce a factor, GDF11, which specifically affects the differentiation/proliferation decisions of the intermediate compartment cells, immediate neuronal precursor, by decreasing their probability to proliferate.

#### Negative regulation of divisions


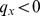
, 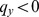. It has been observed that the rate of cell divisions, like the division type, is also under regulation of several types of control loops. For example, in [35] it is stated that in adult neurogenesis, neural stem cell divisions are orchestrated by the mature nervous system environment, and adult-generated neurons and glia appear to be produced on demand, rather than on a fixed schedule. In colon, there is a complex cross-talk of signaling pathways that helps maintain homeostasis. In particular, a negative feedback loop via Lrig1 helps to fine-tune population size and proliferative activity of intestinal progenitor cells [36]. In [26], a “crowd-control” model is described in the context of a local feedback mechanism in the early ventricular zone. Increased densities of neuronal precursors are “sensed” by an increase in the proportion of the cell surface that is occupied by adherens junctions, which leads to a downregulation of hedgehog signaling and results in decreased proliferation [24].

In [33], the quiescence/activation status of stem cells is studied, and it is proposed that both quiescent and active stem cells coexist in stem cell niches, adjacent to each other. The paper studies three different systems, hair, intestine and bone marrow. It is suggested that a negative control loop exists between the active stem cells and quiescent stem cells, which controls divisions of stem cells. It is also hypothesized that a negative regulation is imposed on the quiescent stem cells from the more mature offspring cells. These loops correspond to negative control of stem cell divisions from the stem cell compartment or the differentiated cell compartment respectively.

#### Positive regulation of divisions


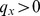
. This mechanism can be hypothesized to be present in any system where a negative control loop (of the type described above) is relatively week. In this case, increasing the number of stem cells will simply lead to an increased net rate of divisions, much like is assumed in e.g. [1], [55], [59] and other models.

#### Division and differentiation decisions may not be independent


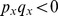
, 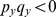. So far we considered the control of senescence/division and proliferation/differentiation decisions as independent. There is however evidence in the literature that they can be intimately connected. In [34], the authors state that there are two fundamental parameters influencing the cellular output of stem cells: (i) their rate of division and (ii) their type of division (differentiation vs proliferation). Recent data suggest that cell cycle length of neural precursors determines not only the first parameter, but also the second one. In [26], [34] it is argued that regulation of the stem cell cycle is related to the regulation of differentiation/proliferation decisions. Studies of the cell cycle of embryonic stem cells have provided evidence that the regulation of G1 is related to the balance between differentiation and self-renewal. The length of the G1 phase corresponds to a window of increased sensitivity to differentiation signals [26], [27], suggesting that a decrease in the division rate is coupled with an increase in differentiation: 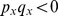, 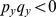. Both Notch and Wnt signaling are important in the regulation of cell divisions, and both have been reported to also play a role in cell fate decisions [34].

### Possible Topologies of Two-compartment Control Networks

As reviewed above, many different combinations of control loops of different signs have been described. Therefore, many different mechanisms can be compatible with stability, as suggested by conditions (4–5). These are some patterns that follow from conditions (4–5):

- There must be at least two control loops in the system.
- These two loops must be associated with two different cell populations. In other words, some sort of control must come both from stem cells and from the differentiated daughter cell population.
- If division and differentiation decisions are coupled as described above 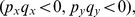 then to ensure the robustness of the regulation, the division rate of the stem cells must be controlled by a negative feedback loop from the stem cell population (e.g. by means of contact inhibition mechanism).
- Negative control of divisions is associated with the largest parameter regions of stability (and thus with highest system robustness). In this case, it is most likely that differentiation is under positive control from the stem cells, and under negative control from the differentiated cell population.

#### Minimal controls

**Figure 2:**
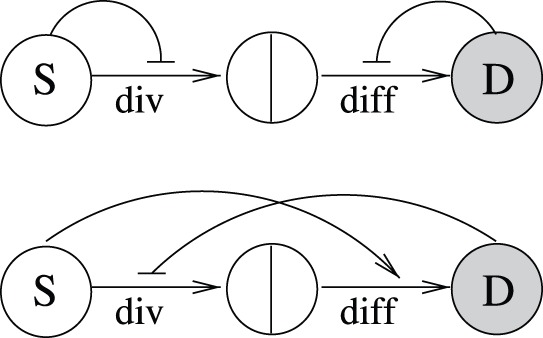
Minimal regulatory networks for a two-compartment system. (a) Networks with two control loops. (b) Networks with three control loops. The circles marked with “S” and “D” denote stem and differentiated cells respectively. The two cell fate decisions are marked as “div” for divisions and “diff” for differentiation. Positive and negative bow-shaped arrows denote control loops.

In accordance with the first two observations listed above, we can identify the most minimalistic control mechanisms compatible with stability. They only include two controls, and are depicted in figure 2(a). There, we present the self-renewing cell lineage as a sequence of two decisions: a division decision of a stem cell, followed by a differentiation decision (which, if positive, results in the production of two differentiated cells). The positive and negative bow-shaped arrows represent control loops. They originate in the respective populations exerting the control. The first minimal control pattern (considered in [58], [61]) contains a negative control of stem cell divisions, e.g. as a result of contact inhibition, and a negative control of differentiation decisions from downstream (the more differentiated cells there are in the system, the less likely the stem cells will be to differentiate). The second minimal control contains a negative regulation of divisions by differentiated cells (which could also be a type of “crowd-control”), and a positive regulation of differentiation by the stem cells. The latter control loop could be a result of a self-renewal-promoting factor being secreted by a stem cell niche, in which case the more stem cells there are, the less likely each of them will self-renew, resulting in a higher differentiating probability.

These two patterns discovered for the two-compartment system play an important role in more general systems, as shown below. Both control mechanisms in figure 2(a) tolerate a limited amount of other dependencies (say, there could be a positive or a negative control loop in the first minimal pattern from the differentiated cell population influencing the divisions decisions, which is still compatible with stability and limited variability). The two control loops depicted however are necessary and cannot be removed.

We can also identify all the control patterns that include exactly three controls, see figure 2(b). The first control pattern in figure 2(b) resembles the one considered in [1], [59]. The other two patterns, to our knowledge, have not been considered theoretically.

#### Analysis of robustness

**Figure 3:**
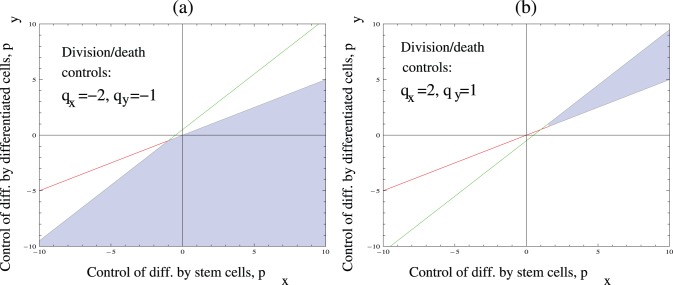
A graphical representation of stability conditions(4–5). For fixed values of controls 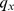 and 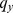, we identify the region of the 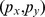 space corresponding to stability of the stem cell system. The borders of this region are given by lines 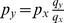 and 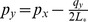. (a) Negative division controls: 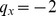, 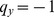. (b) Positive division controls: 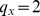, 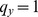. The parameter 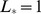.

To explain the last observation listed at the beginning of this section, we note that the two stability conditions (4–5) are imposed in a four-dimensional parameter space 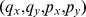 which characterizes the local control of differentiation, death, and proliferation in the vicinity of the fixed point. Let us fix a pair of division controls. For example, let us assume that 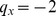 and 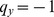 (see figure 3(a)). This means that both the stem cell and daughter cell populations negatively control divisions. Then inequalities (4–5) define a region in the 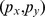 space for which a stable solution is observed (this region is shaded in figure 3(a)). We can see for example that any pair of differentiation controls with 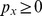, 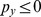 will result in stability. Also, there are relatively large regions with two negative controls (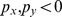) and two positive controls (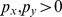). No control with 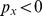 and 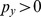 is compatible with stability. Only one negative controls (a downregulation of differentiation by stem or daughter cells) is sufficient for stability in this case (these situations correspond to the 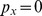, 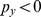 and 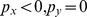 cases).

Figure 3(b) examines a different pair of division controls, 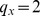 and 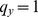. In this case, in order to have a stable solution, parameters 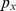 and 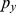 have to belong to a narrow wedge in the first quadrant of the parameter space (this is a consequence of the fact that in this case, 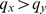). In the opposite scenario, 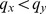, the wedge of stability moves to the third quadrant). It appears that for positive values of 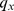 and 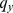, most controls of differentiation are unsuccessful in maintaining stability. In contrast to that, for negative values of 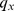 and 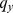 stability can be achieved by a large subset of possible differentiation controls.

To quantify these ideas, we can find the area of the shaded regions in figures 3(a–b), and divide this by the total area of the parameter region considered. Obviously, the area fraction of figure 3(b) is significantly smaller than that of figure 3(a). We can say that the parameter combination of 3(a) is more robust than that of figure 3(b).

**Figure 4:**
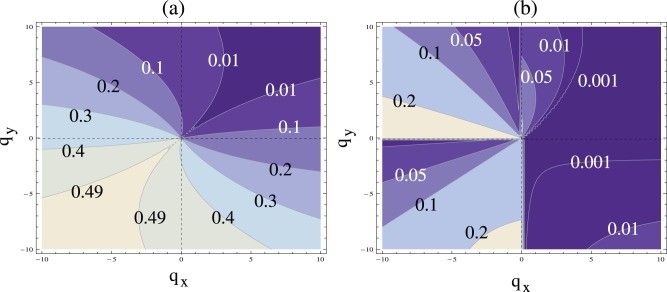
Stability and robustness of two-compartment control systems. (a) For a wide range of positive and negative controls of the division, 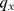 and 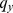, we show how robust the stability of the system is with respect to the choice of the controls of differentiation. The maximum robustness is 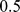 (meaning that if we choose controls of differentiation 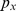 and 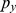 randomly, with probability 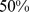 we will get a stable solution). This corresponds to the lightest region in the south-west part of the diagram. The minimum robustness is zero, such that no choice of controls of differentiation will yield a stable solution. This corresponds to the darkest region in north-east part of the diagram. (b) Under the assumption of the connection between division and differentiation decisions, the same contour-plot is shown with the restriction 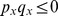, 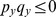. The parameter 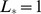.

To investigate robustness in a more systematic fashion, we created figure 4. In the contour-plot of figure 4(a), for each pair 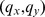, we calculated the fraction of all possible parameters 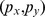 that correspond to a stable solution (this was done by integration over the regions of stability, as explained above). The lighter colors in the contour-plot correspond to larger fractions, and thus to a higher degree of system robustness (in the sense of the word used here). The highest robustness (the area fraction of 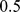) is observed for negative division controls. The lowest robustness (approaching zero) corresponds to positive division controls. Figure 4(b) performs the same analysis under the connectivity conditions 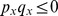, 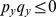.

### Multi-step Systems

**Figure 5:**
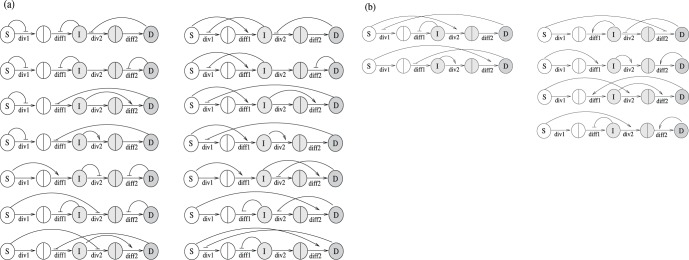
Minimal regulatory networks for a three-compartment system with three control loops. (a) Left: the 7 networks containing (modifications of) the first pattern of figure 2(a). Right: the 7 networks containing (modifications of) the second pattern of figure 2(a). (b) The remaining 6 networks. Notations are similar to those of figure 2, with “I” denoting the intermediate cell type.

Our methods generalize to systems with many intermediate cell types. For example, here we present results for a three-compartment system, consisting of stem cells, transit amplifying cells, and terminally differentiated cells. The following patterns are observed:

- All three populations (stem cells, transit amplifying cells, and terminally differentiated cells) must control at least one process each.
- There must be at least three control loops in the system, which must be associated with three different cell populations.
- The differentiation decision for stem cells, *P* ^(1)^, must be under control from another population. It must be controlled by either a negative loop from downstream, or a positive loop from upstream.
- There are exactly 20 different minimal control types (that is, controls containing exactly three loops), see figure 5. 16 of them have different topology, and 4 more have repeated topology but a different sign arrangement, see the 4 control patterns on the right of figure 5(b). These 20 patterns are the three-compartment equivalent of the two minimal patterns found for the two-compartment model.
- For the minimal control networks, the control of stem cell divisions (from the stem cells, or any of their descendants) must be negative (or zero). This is not the case for the divisions of transit amplifying cells.
- The death rate control of terminally-differentiated cells must be positive (or zero). Each network with a positive control of the death rate can be replaced by an identical network with a negative control of stem cell divisions (thus networks with death-rate control are not included in the 20 minimal networks of figure 4).
- 14 of 20 minimal networks (figure 5(a)) contain elements of the two minimal networks uncovered for the two-compartment system.
- Only 4 minimal networks (depicted on the right of figure 5(b)) contain positive control of differentiation decisions from upstream (the type considered in [1]).

In general, cell lineages may have a large number of intermediate types. For example, [35] studies neurogenesis and identifies the following 5 types of cells (in the order of progressive differentiation) in the subgranular zone of the hippocampal dentate gyrus: radial and horizontal type 1 neural stem cells, early stage type 2a transit-amplifying progenitors, late stage type 3 transit-amplifying progenitors, immature granule neurons, and mature granule neurons. Although the paper discusses a large number of factors involved in the regulation of divisions and differentiation, the exact topology of regulatory loops has not been fully understood. It follows from our analysis that in principle, for such multi-compartment systems, the number of theoretically possible controls grows exponentially. The common patterns listed above, however, can help us single out some of the more likely regulatory mechanisms that may be involved. For example, the first three principles listed above for a three-compartment system, are valid for an 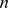-compartment system (with “three” replaced by “
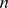
”). Other conditions that can be obtained for the general systems are more technical and can be found in the Materials and Methods section.

## Discussion

In this paper we considered possible mechanisms of control of two- and multi-compartment lineages, and identified general stability conditions for self-renewing tissues. It turns out that even without the exact knowledge of the functional forms, it is possible to gain a lot of information simply based on the (i) topology and (ii) signs of the control loops. We identified several very general conditions on control networks necessary for a stable maintenance of homeostasis, and further we were able to calculate the variance experienced by cell populations.

Our approach differs from many papers published in the past in two ways. Firstly, unlike most of the theoretical papers, we do not consider any specific functional dependencies, but instead employ the method of axiomatic modeling. On the one hand, as demonstrated in the review of experimental literature presented here, only some of the dependencies are starting to be discovered in a qualitative way (and not in a way that can be translated into a specific function). On the other hand, as seen from the literature, many different types of control loops are in principle possible. Therefore, we examine the whole wealth of possibilities, and single out what components are necessary, and which ones are likely in a biological system.

Secondly, our conceptual approach is also different from the one taken by most experimental groups. Instead of concentrating on “within-cell” regulatory networks, we focus on the “population biology” of stem cell lineages. That is, we are interested in the behavior of different compartments as they respond to the changes in other compartments. This approach is of course not sufficient on its own, but only in addition to the traditional approaches. In order to utilize our methods, we have to use the knowledge accumulated by the studies of individual cellular responses to various factors. We claim however that our approach can be very useful in combination with the traditional methodologies, as it helps to build the “big picture” of the whole lineage behavior, as it is generated by all its different parts.

As a consequence, we hope that this paper helps initiate a line of experimental research with a slightly shifted emphasis. As of now, it is relatively clear what role different factors play in cell fate regulation, and how they shape cell fate decisions. It is often unclear however, where these factors come from. In other words, they are usually investigated by either adding them to the system exogenously, or blocking their action by mutations. It is often unknown which cell populations are mostly responsible for generating these factors. With this information available, one will be able to reconstruct not just within-cell regulatory loops, but also the inter- and intra-compartment control loops studied here, which enables us to understand the lineage as a whole, as a self-regulating mechanism which robustly maintains the system near its equilibrium.

Finally, we list modeling assumptions which necessarily restrict the applicability of the current work, and also provide avenues for further developments. Our model is fully-stochastic, but it does not take explicit account of spatial effects. Implicitly, some of the negative control loops studied here can be interpreted as having spatial origins. A more systematic study of spatial constraints however remains a challenge. Furthermore, the theory developed in this paper is only valid near the equilibrium state of a self-renewing system. In other words, our models cannot be extended to the situations where the tissue is recovering from a severe injury, or is developing from a small number of stem cells. Our analysis is based on the “local” behavior of controls (that is, their derivative at equilibrium). For such large deviations from the equilibrium, more information is needed about the control functions and their behavior far from the equilibrium. Finally, the current model does not include phenomena such as de-differentiation and asymmetric divisions, which have been reported to occur in various healthy and cancerous tissues.

## Materials and Methods

### Stochastic Two-step Model

#### Model formulation

Suppose the population consists of 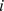 stem cells and 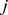 daughter cells. The stem cells divide at a rate 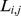. Upon division, with probability 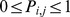 two daughter cells are produced; this represents the process of differentiation. With probability 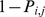 two stem cells are produced, representing the process of stem cell proliferation. Finally, the daughter cells die at a rate 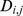. Let us denote by 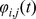 the probability to have 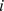 stem cells and 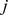 daughter cells in the population at time 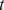. The Kolmogorov forward equation is then given by the following:
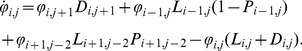
(6)


Here, the first term on the right hand side is the death of daughter cells, then proliferation of stem cells, differentiation, and finally, the possibility of no change.

Let us introduce the operators 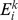 and 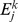, such that
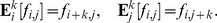



Then equation (6) can be rewritten more conveniently,
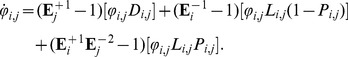
(7)


#### The linear noise approximation

Equation (7) is nonlinear, and a general solution cannot be found. Therefore, we will use approximate methods to solve it. Let us assume that the functions 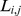, 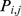, and 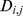 depend weakly on their arguments:
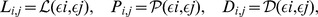
where 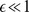. We will use this parameter to perform the Van Kampen master equation expansion, in order to formulate the linear noise approximation [62]. We expect that in the long run, the probability distribution, 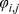, will have a peak somewhere around the (large) values



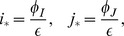
with 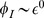, 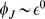. Let us suppose that the width of those peaks scales with 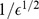. This is expressed in the following change of variables,



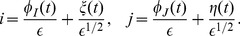
(8)This change of variables will be used in the master equation (7). First of all, the probability function 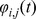 is now a function of 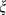 and 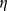:
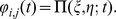



Its time-derivative can be written as follows,
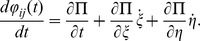



Because the left hand sides of expressions (8) are time-independent, we have 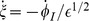, 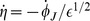. Also, we will introduce a slow time-scale,
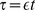
(the necessity for this rescaling will become apparent once all the terms at different orders of 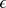 are collected in the master equation). Therefore, we have for the time-derivative of 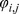:



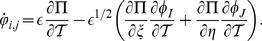
(9)Next, we evaluate the shift operators. A jump of size 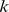 in the value of 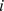 is reflected by the jump of size 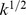 in the value of 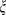:
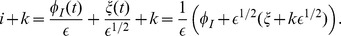



Similar arguments hold for the values of 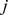. This allows us to express the shift operators 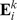 and 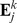 in terms of a (Taylor) series of differential operators,
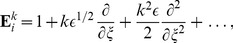
(10)and similarly for the shift in the 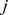-direction.

Finally, we use ansatz (8) to expand the functions 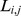, 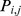, and 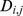. We have
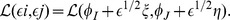



It is convenient to denote 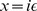, 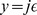, such that 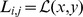, and denote by the subscripts the derivatives of this function with respect to its argument: 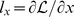, 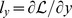, etc. We have
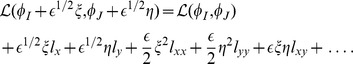



Similarly, we expand the functions 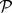 and 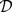. These expressions, together with the operator expansions (10) and the time-derivative (9), are substituted into the master equation (7). Then the terms in different orders of 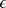 are equated. At order 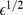 we have
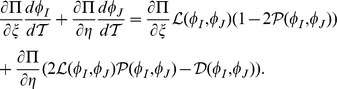



This equation gives rise to two “macroscopic laws”,
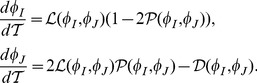
(11)or in steady state simply



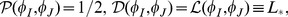
(12)see equations (3) with 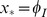, 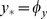. Let us introduce the notations
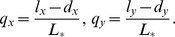



Linear stability of solution (12) can be investigated by standard methods and gives rise to conditions (4–5).

Next, at order 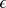 of the master equation expansion, after rescaling time once more by
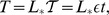
we obtain the following linear Fokker-Planck equation:



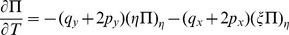


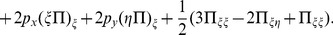
(13)


This is the linear noise approximation of Van Kampen [62]. The validity of this approximation has been studied extensively, see e.g. [63], [64]. Here we mention that the relative size of typical fluctuations scales with 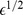, and thus for sufficiently small values of 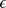, the system will remain near the equilibrium and stochastic extinction is an unlikely event, at least for a time-duration which grows with 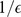. For a rigorous study of extinction times of birth-death processes see e.g. [65], [66].

From equation (13) we can obtain the equations for the first and second moments in a standard way:
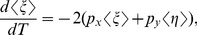
(14)

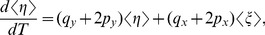
(15)

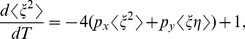
(16)

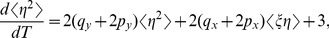
(17)

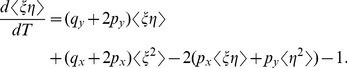
(18)


The solution of this system yields
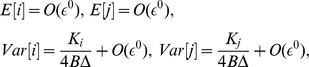
where



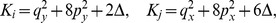
such that the variance expressions have contributions in the order 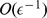, consistently with our original assumption (8). We notice that if the fixed point is stable, then 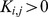, and conditions (4–5) become conditions of the positivity and finiteness of the variance of the two cell populations. In particular, for tighter control of the variance, one should make the quantities 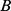 and 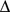 as large as possible.

### Deterministic Description

#### Stability

A deterministic description of this problem is given by equations (1–2) (or (11). The Jacobian corresponding to solution (3) is given by constant 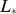 multiplied by the matrix
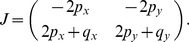



The negativity of the eigenvalues requires exactly the condition (4–5) to be satisfied. Of course, this analysis does not inform us about the populations fluctuations, therefore we performed the stochastic analysis to gain insights into the regulation of the variance.

It is instructive to put our analysis in the context of the previous studies of similar systems. In [43], [45], both local and a global stability analysis was performed. Under specific assumptions on the global behavior of the regulation functions, the complete phase portrait of the behavior was obtained. Analysis of the structure of stationary solutions in the 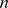-compartment version of the model was presented in [44].

In this paper, we only present local, linear stability analysis of the equilibrium number of cells. The corresponding results of [43], [45] can be viewed as a special case of the general conditions (4–5). On the other hand, papers [43], [45] go a lot further in their analysis because they study global stability of the underlying systems. This of course requires the knowledge of the global behavior of the regulatory functions. In this paper we concern with the local analysis only (and thus all the results only depend on the behavior of the controls at the equilibrium point). A global stability analysis would require an imposition of further conditions of the control functions and lies beyond the scope of the current study.

#### Robustness

The equilibrium values of 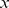 and 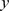 are defined by equations (3). We would like to determine how these values depend on the model parameters (the functions 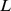, 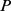, and 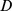). Consider a point 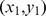 in the vicinity of 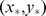. At this point, equations (3) can be rewritten by expanding the functions in the Taylor series around the equilibrium point:
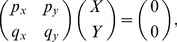
(19)where we denoted 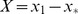, 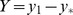. Let us denote by 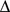 the determinant of the matrix on the left hand side of this equation, which is consistent with definition in (5). As long as 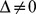, equation (19) admits only the trivial solution, which again reiterates the fact that the point 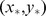 is the equilibrium. Now, let us suppose that the controls changed such that the rate of divisions is now 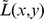 and the probability of differentiation is 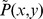; their Taylor expansion coefficients are also denoted by tildes. Now we have the equation



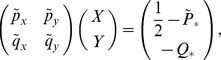
where 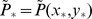 and 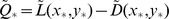. The solution 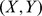 is the change in the equilibrium point as the result of the change in the functions 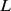 and 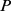. It is clear that the values of 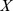 and 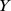 are inversely proportional to the matrix determinant, 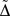. Therefore, to ensure the robust behavior of the system, we need to increase the absolute value of the determinant. The closer it is to zero, the less robust the system is with respect to parameter changes.

### Multiple Intermediate Compartments

Suppose we have a cascade of differentiating cells, 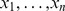, where 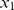 corresponds to stem cells, each subsequent class is characterized by a smaller degree of “stemness” and a higher degree of differentiation, and 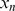 corresponds to terminally differentiated cells. Each class 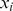 with 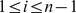 has the rate of divisions given by 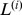, and the probability of differentiation 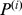. The class 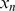 has the death rate 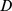. The quantities 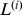, 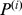, and 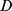 are in general functions of all the variables, 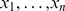. We can write down the deterministic system describing the dynamics of these cell classes:
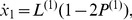
(20)

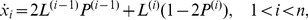
(21)

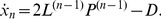
(22)


Let us consider the steady state, 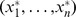, and denote the values of all the rates at the steady state as 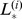, 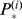 and 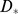. At steady state, we have the following equations:
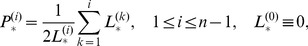
(23)

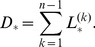
(24)


In particular, at steady state we have 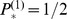. Let us introduce the following rescaled quantities:
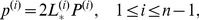
(25)

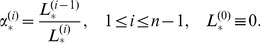
(26)


Here, quantities 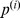 are functions of all the variables, 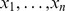, and 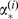 are constants. With these new notations, the Jacobian of system (20–22) is given by 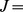


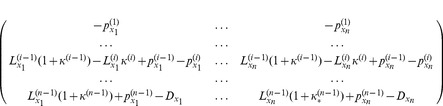
(27)where the coefficients are defined as



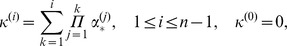
and the subscripts 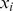 denote partial differentiation with respect to 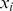.

A necessary condition for the negativity of all the eigenvalues of 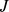 is that 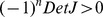. The nonzero value of 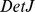 is also the condition for the robustness with respect to parameter changes, which follows from a direct generalization of the argument given for a two-compartment system.

From condition 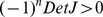 it follows that at least one of the derivatives of 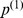 must be nonzero. In other words, there must be control of the stem cell differentiation rate.

Next, since all the terms are derivatives, we need to require that there is at least one nonzero derivative in each column. This means that each of the populations, 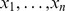 must influence at least one of the processes (division, differentiation, or death). In other words, there cannot be a population that does not influence at least one of the rates.

Let us suppose that we have exactly one nonzero derivative with respect to each of 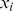. Then we claim that the functions exhibiting dependence on all these variables must all be different functions. In other words, there cannot be for example 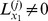 and 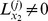.

Finally, it follows that there cannot be two consecutive populations with no control on any of their functions. It is possible to have a population whose rates are not controlled, but there must be some control on the processes of the populations immediately upstream and downstream from it.

### Minimal Controls

We ask the question: can we identify, biologically and mathematically, qualitatively different types of control? We approach this question by finding minimal sets of requirements for stability. For the two-compartment model, we note that at least two of the four quantities, 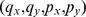, must be nonzero to satisfy condition (5). In fact, there are exactly two cases where only two of the four derivatives are nonzero, see figure 2:

[1] 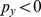 and 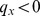.

[2] 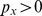 and 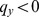.

For these two cases, the two remaining derivatives can be zero, or they can have a (possibly limited) magnitude of either sign, such that the system is still compatible with conditions (4–5). No other logical possibility of controlling the system with only two nonzero variables exists.

At the next level of complexity, we consider the possibility of having three nonzero controls. There are 3 such cases, see figure 2(b):

[3] 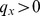, 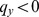, and 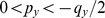.

[4] 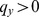, 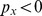, and 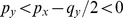.

[5] 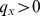, 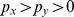.

Cases [3]–[5] are defined according to the following rules: (i) they have three nonzero controls, and (ii) they cannot be reduced to cases [1]–[2] by simply setting one of the controls to zero. For example, it is possible to set a stable system of controls by arranging 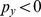, 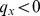, 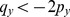, but in this case setting 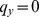 reduces this to case [1]. Each of the cases [3]–[5] constitutes a totally different type of control, where having all three components of control with the given signs and restrictions is necessary. Adding the fourth nonzero derivative of a limited magnitude of either sign will still be compatible with conditions (4–5).

In the case of a three-compartment model, stability conditions have a more complicated form. They correspond to the negativity of the real part of the eigenvalues of Jacobian (27), which has dimensions 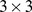. The conditions can be obtained by using the Routh-Hurwitz stability criterion. The results pertaining to the signs of controls are shown in figure 5.
